# Supplementary material for: Green Synthesis of Cobalt-Doped CeFe2O5 Nanocomposites Using Waste Gossypium arboreum L. Stalks and Their Application in the Removal of Toxic Water Pollutants
Source: Nanomaterials (Basel). 2024 Aug 12;14(16):1339. doi: 10.3390/nano14161339 (PMC11357551; doi:10.3390/nano14161339)
Supplement: Supplementary file 1 [file nanomaterials-14-01339-s001.zip › nanomaterials-2964164-Supplementary Materials.pdf]

Supplementary Materials

Green Synthesis of Cobalt-doped $CeFe_2O_5$ Nanocomposites Using Waste *Gossypium arboreum* L. Stalks and their Application in the Removal of Toxic Water Pollutants

Saloni Koul [1], Mamata Singhvi [1,*] and Beom Soo Kim [2]

[1] Department of Biotechnology (with Jointly Merged Institute of Bioinformatics and Biotechnology), Savitribai Phule Pune University, Pune 411007, India; n20502022@uopca.unipune.ac.in
[2] Department of Chemical Engineering, Chungbuk National University, Cheongju 28644, Chungbuk, Republic of Korea; bskim@chungbuk.ac.kr
* Correspondence: mamata.singhvi@campstud.unipune.ac.in; Tel.: +91-20-25690442

S1. Materials and methods

S1A. Biochemical analysis of cotton stalk waste

The compositional content of cotton stalk was determined through the protocols of the standard association of analytical communities (AOAC) method as reported previously [1,2].

S2. Results and Discussion

S2A. Biochemical analysis of cotton stalk waste

Lignocellulosic biomass, serves as substrates for various application including green synthesis of nanomaterials which depends primarily on carbohydrate and lignin content. As shown in Table S1, the raw cotton stalk biomass used in this study contained large amounts of cellulose (43.2 %, w/w) and hemicellulose (23.2 %, w/w). The lignin content of the biomass was found to be 28.9 %, w/w, which releases phenolic compounds during the extraction process. The released phenolic and aromatic functional groups obtained during thermal extraction of cotton stalks aids in developing functional NCs.

Table S1. Biocompositional analysis of raw cotton stalk waste powder.

Biomass Components	Composition (%)
Cellulose	43.2
Hemicellulose	23.2
Lignin	28.9
Ash	0.31
Extractives	4.21
Total	8

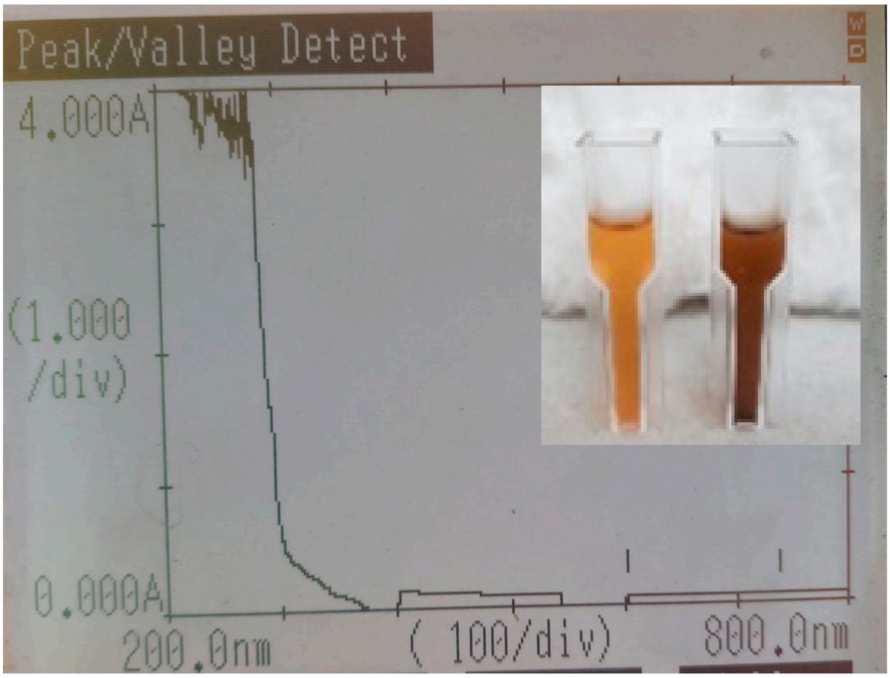

Figure S1. UV-Vis spectrometry analysis of synthesized CCIO NCs.

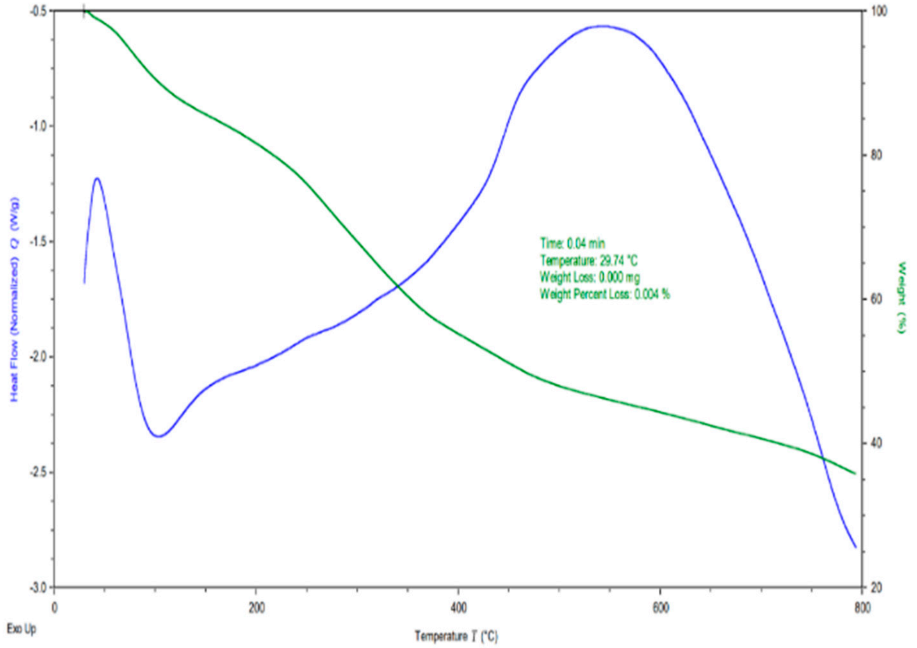

Figure S2. TGA analysis of synthesized CCIO NCs.

References

1. Singhvi, M.S.; Deshmukh, A.R.; Kim, B.S. Cellulase Mimicking Nanomaterial-Assisted Cellulose Hydrolysis for Enhanced Bio-ethanol Fermentation: An Emerging Sustainable Approach. *Green Chem.* **2021**, *23*, 5064–5081, doi:10.1039/D1GC01239H.
2. Rajak, R.C., Saha, P., Singhvi, M., Kwak, D., Kim, D., Lee, H., Deshmukh, A.R., Bu, Y. and Kim, B.S., An eco-friendly biomass pretreatment strategy utilizing reusable enzyme mimicking nanoparticles for lignin depolymerization and biofuel production. *Green Chem.* **2021,** *23*, 5584-5599.
